# South African Speech‐Language Therapists’ Perceptions of Youth Offenders and Youth Offender Rehabilitation

**DOI:** 10.1111/1460-6984.70345

**Published:** 2026-09-30

**Authors:** Johan Jacobus Maasz, Christiaan Bezuidenhout, Marien Alet Graham, Jeannie van der Linde

**Affiliations:** ^1^ Department of Speech‐Language Pathology and Audiology University of Pretoria Pretoria South Africa; ^2^ Department of Social Work and Criminology University of Pretoria Pretoria South Africa; ^3^ Department of Mathematics Education University of Pretoria Pretoria South Africa

**Keywords:** perceptions, rehabilitation, scope of practice, speech‐language therapists, survey research design, youth offender, South Africa

## Abstract

**Background:**

Healthcare professionals (HCPs) tend to display similar levels of implicit bias towards certain groups as the general population. This is concerning as such biases are known to result in poorer clinical decision‐making and therapeutic outcomes for offender populations. Understanding the perceptions that HCPs hold towards the groups they serve are therefore essential. Given the promising role that speech‐language therapists (SLTs) could fulfil in youth offender rehabilitation, their perceptions towards the youth offender population needs further exploration. This study explored South African SLTs’ perceptions of youth offenders and youth offender rehabilitation.

**Methods and procedures:**

Using non‐probability purposive sampling, a cross‐sectional, mixed‐method online survey was distributed on social media platforms. Thirty‐five SLTs registered with the Health professions council of South Africa (HPCSA) and currently practising in South Africa volunteered their participation. Quantitative data were analysed using point‐biserial and Spearman correlations, while qualitative data were analysed using qualitative content analysis.

**Results:**

Most participants had not received training related to working with individuals in the criminal justice system (CJS) [*n* = 32, 91.4%] and had not previously delivered services to youth offenders in the CJS (*n* = 25, 71.4%). More than half of the participants agreed that youth offenders can be rehabilitated and functionally reintegrated after incarceration (*n* = 21, 60.0%). The majority (*n* = 29, 90.3%) also agreed that societal attitudes contribute to the challenges that youth offenders face during reintegration. Participants reported that communication disorders could negatively influence the behaviour and social interactions of youth offenders by contributing to emotional and behavioural difficulties. Participants with no previous training in working with persons in the CJS were more likely to agree that biological risk factors such as a genetic predisposition (*p* = 0.048, *r_s_* = 0.358) and a diagnosis of Autism Spectrum Disorder (*p* = 0.016, *r_s_* =  0.430) could contribute to youth offending behaviour.

**Conclusions and implications:**

Participants generally perceived youth offenders and their potential for rehabilitation positively. They also demonstrated good insight into potential barriers and societal attitudes that affect youth offender rehabilitation and reintegration. Additionally, participants without prior training to work with youth offenders were more likely to attribute criminal behaviour to personal and biological factors than participants who had received such training. Further research exploring HCPs' perceptions of youth offenders across different disciplines and contexts is recommended.

**WHAT THIS PAPER ADDS:**

*What is already known on this subject*

- Youth offenders are at greater risk of developing communication difficulties. Subsequent research has focused on examining the prevalence and characteristics of the speech, language and communication needs (SLCN) within this population. In addition, a small number of preliminary studies have explored the efficacy of speech‐language therapy (SLT) interventions for the population. What remains largely unexplored, however, is how SLTs perceive this population, despite evidence that such perceptions can shape clinical decision‐making and patient engagement among healthcare professionals (HCPs).

*What this study adds to the existing knowledge*

- This study offers preliminary insights into South African SLTs’ perceptions of youth offenders, their rehabilitation, and the social attitudes that may influence the rehabilitation process. It also highlights the biological, environmental, and mixed risk factors that participants identified as potential contributors to youth offending behaviour.

*What are the clinical implications of this study?*

- Although this study has no direct clinical implications, its findings may be considered by SLTs in youth rehabilitation and could encourage HCPs to reconsider how they access and assess detained youth offenders. It could also inform public health policies on treatment modalities for youth offenders. Finally, this study provides a foundation for further research on HCPs’ perceptions of justice‐involved youth.

## 1 Introduction

Healthcare professionals (HCPs) must deliver fair, just, and equitable, evidence‐based care to individuals from diverse sociocultural and demographic backgrounds (Campbell 2025). Unfortunately, research has shown that HCPs typically display levels of implicit bias towards certain groups comparable to those observed in the general population (FitzGerald and Hurst 2017; Maina et al. 2018), which can influence clinical decision‐making and patient engagement (Sarteschi 2013; Skinner and Farrington 2023). Similarly, research on correctional healthcare workers, such as nurses, has found that their perceptions of offender populations are mainly shaped by primary socialisation and professional experience (Hardesty et al. 2007). Understanding how HCPs perceive the populations they serve is therefore essential, as their perceptions are often shaped by implicit biases and stigma (Chapman et al. 2013; Zestcott et al. 2016).

Research demonstrates that HCP attitudes, whether implicit or explicit, influence clinical judgement, affect interpersonal interactions, and ultimately influence therapeutic outcomes (Chapman et al. 2013; Zestcott et al. 2016). In the context of criminal justice, recent research revealed that HCPs often hold negative attitudes toward offender populations (Martinaki et al. 2025; Vorstenbosch et al. 2025). This may translate into disparities in care, youth offenders disengaging from services, and reduced rehabilitative success (Bui and Deakin 2021; Veilleux et al. 2022). Concerns of this nature are especially pressing in correctional settings, where the stakes of therapeutic failure are high—not only for individuals but also for communities, due to the implications for recidivism and reintegration (Adjorlolo et al. 2016; Martinaki et al. 2025). Youth offenders^1^ represent a notably vulnerable cohort among the offender population (Ahmed Shafi 2020). Compared to their non‐offending peers, youth offenders experience higher rates of complex and comorbid physical, mental, and developmental health conditions (Lennox et al. 2014). Among these, speech, language, and communication needs (SLCNs) are exceedingly prominent, with research consistently documenting higher rates of communication difficulties in this population (Chow et al. 2022). These needs are concerning given their strong association with youth offending behaviours (Chow et al. 2022) and adverse long‐term outcomes, including academic underachievement, behavioural challenges, and social isolation (Langbecker et al. 2020; Lloyd‐Esenkaya et al. 2021). These factors, in turn, are known predictors of continued offending behaviour and recidivism (García‐Gomis et al. 2017; United Nations Office on Drugs and Crime [UNODC] 2012). Additionally, these difficulties could negatively affect the participation of youth offenders in rehabilitation programs geared towards preventing reoffending (Bryan et al. 2015; Winstanley et al. 2020), further increasing the risk of recidivism in the population. Studies conducted in the United Kingdom (Bryan and Gregory 2013), New Zealand (Howard et al. 2020), and Australia (Reid et al. 2025; Snow et al. 2018) have found that professionals working within youth justice systems recognise both the prevalence of SLCNs among youth offenders and the importance of speech‐language therapists (SLTs) in providing services within these settings.

SLTs are uniquely positioned to address the SLCNs of youth offenders, as their specialised knowledge and experience in assessing and treating communication difficulties enable them to play a vital role in supporting the cognitive, social, and emotional development of justice‐involved youth (Anderson et al. 2022; Zupan et al. 2022). Despite this, youth justice settings are not currently a service delivery context that SLTs in South Africa are involved in, resulting in limited service provision for this population. Student SLTs are also not currently trained in these settings. DCS prioritises general medical, nursing and psychological services, while access to specialised services such as SLT is largely dependent on referrals to the public healthcare system (DCS 2005). Accordingly, the DCS's most recent *Annual Report* makes no reference to the provision of SLT services for offenders (DCS 2025). Consequently, SLTs who have provided services to youth offenders have typically done so through public hospitals and schools rather than within youth justice settings resulting in limited experience with this context (Maasz et al. 2026). While existing studies have established the prevalence of SLCNs in incarcerated youth populations (Bryan et al. 2010; Fitzsimons and Clark 2021; Hughes et al. 2017b; Kippin et al. 2018, Kippin et al. 2021; Shirley et al. 2024; Swain et al. 2020) and provided preliminary evidence supporting the efficacy of SLT intervention in this context (Gregory and Bryan 2011; Snow and Woodward 2016; Swain et al. 2020), there is a notable dearth in literature regarding SLTs’ perceptions of youth offenders and their rehabilitation.

This lack of evidence is particularly significant given the broader literature linking HCP bias to disparities in treatment outcomes (Campbell et al. 2025). Considering the promising role that SLTs could fulfil in youth offender rehabilitation, their perceptions towards the population need further exploration. Accordingly, this study explored *South African SLTs’ perceptions of youth offenders and youth offender rehabilitation*.

## 2 Method

A cross‐sectional, mixed‐method open online survey design using non‐probability purposive sampling was used. IRB clearance was obtained (HUM005/0324).

### 2.1 Study Population and Sampling

The study targeted qualified SLTs registered with the Health Professions Council of South Africa (HPCSA) and actively engaged in professional practice within the country. Active practice was defined as participation in discipline‐relevant roles, including clinical service provision, teaching, and supervision. Given the lack of SLT services within South African youth justice settings, all South African SLTs were eligible for inclusion in the sample. Restricting participation to SLTs with experience in youth justice settings specifically would have limited the ability of the researchers to obtain insight into how SLTs view youth offenders and their rehabilitation. An a priori power analysis conducted using G*Power (Faul et al. 2007) was used to determine the sample sizes required to detect effects of varying magnitudes at a statistical power of 0.80. The analysis indicated that a minimum sample of 782 participants would be required to detect a small effect size (0.10), 84 participants would be required to detect a medium effect size (0.30), whereas a sample of 29 participants would be sufficient to detect a large effect size (0.50). Many researchers have suggested that it's unnecessary to obtain the minimum sample size required to detect small effect sizes, as finding a statistically significant result for a small effect may have statistical significance (*p* < 0.05), but not real‐world or practical significance (Baicus & Caraiola 2009; Peeters 2016). Larger effect sizes can generally be detected with smaller samples at the same level of statistical power. Accordingly, the study aimed to recruit at least 84 participants to enable the detection of medium‐sized effects. However, recruitment was limited by the relatively small population of South African SLTs, the specialised nature of the topic, and the fact that participation was voluntary. Consequently, only 35 eligible participants were recruited during the data collection period. Because the final sample consisted of 35 participants, the study had adequate statistical power only to detect relatively large effects. Consequently, smaller associations may not have been detected even if they were present and the findings should therefore be interpreted as preliminary.

Out of 44 individuals who initiated the online survey, one response was excluded for not meeting the inclusion criteria, and eight incomplete responses were removed, leaving 35 responses for analysis. The responses of the eight respondents that were excluded primarily completed only the initial biographical questions and did not proceed to the substantive survey items related to the study phenomenon. Several of these responses included only one or two biographical items before the survey was discontinued, resulting in insufficient data for meaningful inclusion in the analysis. All respondents (*n* = 35, 100%) were registered with the HPCSA and practising within South Africa at the time of their participation. Participants ranged in age from 22 to 74 years (*M* = 35.14, *SD* = 13.47), and their professional experience varied from one to 52 years (*M* = 14.40, *SD* = 13.37). A majority of participants identified as female (*n* = 33, 94.3%) and two identified as male (5.7%). In terms of academic qualification, 17 participants (48.6%) had completed a four‐year qualifying bachelor's degree, 13 (37.1%) held master's degrees, 3 (8.6%) held doctoral qualifications, and 2 (5.7%) had completed postdoctoral research fellowships. The demographic characteristics of the sample are broadly representative of the South African SLT workforce. Nationally, the profession is characterised by a relatively young workforce, with 48% of SLTs aged between 25 and 35 years, while males constitute a minority of practitioners (Pillay et al. 2020). Additionally, although limited data are available on the distribution of undergraduate and postgraduate qualifications among South African SLTs, a four‐year professional bachelor's degree from an accredited South African university is the minimum qualification required for registration with the HPCSA. It is therefore unsurprising that most participants reported holding only an undergraduate qualification.

### 2.2 Materials and Data Collection

Data was collected using an online survey administered through the Qualtrics XM platform. The survey, tailored to reflect the South African context, comprised 16 closed‐ended items (including categorical and Likert‐scale questions) and 12 open‐ended questions designed to elicit detailed responses. The survey consisted of three sections: demographic information, perceptions regarding youth offenders and youth offender rehabilitation, and perceptions regarding the possible role of SLPs in youth offender rehabilitation (Supplementary Materials). The survey questions were designed to suit the thematic focus of the survey. The lack of existing literature on the topic resulted in the survey being developed inductively based on the research objectives. The risk factors selected for inclusion in the survey were based on biological, environmental and mixed risk factors typically associated with language disorders in children. The authors selected to investigate the participants’ perceptions of the risk factors which might contribute to youth offending, as these may provide insight into how participants conceptualise youth offenders and understand the potential causes of youth offending behaviour. The average completion time was approximately 10 to 12 min. Survey questions were presented in a linear order to all participants using adaptive questioning. Participants could review their questions by using a “back” button. Due to the large volume of data collected from the survey, the results were reported in two separate and more focused articles. The first study entitled “*Exploring the self‐perceived role of South African speech‐language pathologists in youth correctional facilities*” has been published in the American Journal of Speech‐Language Pathology (Maasz et al. 2026).

To establish content validity, the survey underwent expert review using the Content Validity Index (CVI) methodology described by Yusoff (2019). The CVI is a quantitative measure used to assess the extent to which individual items are relevant and representative of the construct being measured. It is calculated by asking expert reviewers to rate each item (typically on a 4‑point scale), after which the proportion of experts who rate an item as relevant (usually scores of 3 or 4) is computed. An overall CVI is then obtained by averaging the item‐level CVIs across all items. Four professionals—two with doctoral degrees in Speech‐Language Pathology, one with a PhD in Criminology, and one holding a Master's degree in Speech‐Language Pathology evaluated the instrument. Collectively, the reviewers offered 95 years of relevant experience. The final version obtained a CVI of 1.0, indicating a strong content validity given the expert panel size (Yusoff 2019).

Survey hyperlinks and infographics were distributed via profession‐specific Facebook groups, other social media platforms such as LinkedIn, and through an email circular to members of the South African Speech‐Language‐Hearing Association (SASLHA). Participants volunteered their participation. Before gaining access to the survey, participants were required to review and accept an informed consent form. Respondents had access to the survey for five weeks, and no financial or material incentives were offered for participation. No identifying information was collected from participants, and the survey was preset not to collect individual Internet Protocol (IP) addresses from participant devices.

### 2.3 Data Analysis

Quantitative data were analysed using IBM SPSS Statistics (version 30) at the 5% significance level. Descriptive and inferential statistics were used to summarise findings and explore relationships among variables. In previous literature, socio‐demographic variables such as age (Adjorlolo et al. 2016; Martinaki et al. 2025), years' experience (Adjorlolo et al. 2016; Martinaki et al. 2025), level of education (Martinaki et al. 2025), and a history of service delivery (Hardesty et al. 2007) have been found to influence HCP's attitudes towards offenders. Correlations were therefore conducted to examine these relationships. In addition to these, exploratory correlations were conducted to examine the relationships between training in working with persons in the CJS and the belief that youth offenders can be rehabilitated and functionally reintegrated into society.

Point biserial correlations (r_pb_) were used to assess relationships between binary and ordinal variables, while Spearman correlations (r_s_) were used for associations involving two ordinal variables or a combination of ordinal and continuous variables. Pairwise deletion was employed to address missing values, as it preserves more data points than listwise deletion and avoids the loss of entire data sets when only a single value is missing (Mirzaei et al. 2022). Formal adjustments for the familywise error rate, such as Bonferroni correction, were not applied. These procedures control the probability of Type I error across multiple comparisons; however, they make the interpretation of results contingent on the number of tests performed, introducing arbitrary variability—such that two researchers examining the same hypothesis could reach different conclusions based solely on how many comparisons they conduct (Perneger 1998). Furthermore, Bonferroni‐type adjustments are widely regarded as overly conservative, substantially increasing the risk of Type II errors and potentially obscuring meaningful associations that warrant further investigation (Barnett et al. 2022). As noted by Perneger (1998), routine application of such corrections may also lead to inefficient research practices and undermine comprehensive analyses. Accordingly, all correlation analyses are reported with exact p‐values and interpreted cautiously rather than being mechanically adjusted for the familywise error rate.

Qualitative responses were analysed using inductive qualitative content analysis as described by Vaismoradi et al. (2013). Participant responses were broken down into sentences, and codes were derived using participants’ wording. These codes were then grouped into broader categories or themes. Where possible, the frequency of recurring themes was reported to highlight common patterns across responses.

## 3 Results

Participant demographic information is presented first, followed by participant perceptions of youth offenders and youth offender rehabilitation.

### 3.1 Education and Training in Working with Youth Offenders

Only a small proportion of participants reported receiving training related to working with individuals within the CJS; three respondents (8.6%) reported such training, while the remaining 32 respondents (91.4%) indicated they had not. Among those who had received training, it was primarily informal (*n* = 2, 66.7%), while one participant (33.3%) reported holding a Bachelor's degree in criminology in addition to their qualification in SLT.

Most respondents (*n* = 25, 71.4%) reported not having delivered SLT services to youth offenders involved in the CJS. A smaller number (*n* = 6, 17.1%) indicated that they had, and four participants (11.4%) were uncertain whether they had. All six participants who confirmed working with youth offenders noted that their service provision to this population was infrequent (*n* = 6, 17.1%). These services were most commonly delivered in hospital settings (*n* = 4, 66.7%) but also took place in correctional facilities (*n* = 1, 16.7%) and schools (*n* = 1, 16.7%). The most frequently cited age groups of youth offenders served were 24 to 25 years (*n* = 4, 66.7%), followed by 14 to 15 years (*n* = 3, 50.0%), 22 to 23 years (*n* = 2, 33.3%), and 16 to 17 years (*n* = 2, 33.3%). Fewer participants reported working with youth offenders aged 18 to 19 years (*n* = 1, 16.7%) and 20 to 21 years (*n* = 1, 16.7%).

### 3.2 SLTs' Perceptions of Youth Offenders and Youth Offender Rehabilitation

More than half of the participants agreed that youth offenders can be rehabilitated and functionally reintegrated into society (*n* = 21, 60.0%), while four participants (11.4%) disagreed with the statement, and 10 participants (28.6%) were unsure. Participant age (*p* = 0.511, *r_s_* =  −0.115), years’ experience (*p* = 0.332, *r_s_* =  −0.173), level of education (*p* = 0.875, *r_s_* =  0.028), and history of training to work with persons in the CJS [*p* = 0.240, *r_pb_* = 0.204], and a history of service provision to youth offenders (*p* = 0.510, *r_pb_* = 0.123) did not appear to influence this belief. When asked why they believed that youth offenders could or could not be rehabilitated and reintegrated into society, four distinct themes emerged (Table 1).

**TABLE 1:** Qualitative content analysis of participant beliefs about youth offenders’ capacity for rehabilitation and reintegration (*n* = 35).

Code	n	Participant quotes
*Theme 1: Youth offenders’ inherent potential for rehabilitation*		
Developmental stage and maturity level	7	P5: *“Adolescence is a time when the human brain is still developing, especially the prefrontal cortex… this implies that young people might be less likely to have established harmful practices and more open to change.”* P16: *“Youth are often immature, or performing criminal offences due to lack of knowledge or impulsivity control…”*
SLT participants' beliefs	5	P9: *“I have hope when people are shown and taught something better that they are innately good.”* P29: *“I do think each person deserves the opportunity to be supported towards rehabilitation.”*
Crime is driven by context	3	P3: *“I think youth offenders are products of their circumstances and with support can adjust their approaches.”* P16: *“Youth are often… being influenced by others older than them.”*

*Theme 2: Systemic barriers influencing rehabilitation and reintegration*		
Lack of resources and reduced system functionality	5	P2: *“South Africa has an unfair distribution of funds, resources and support.”* P20: *“The current state of resources available in South Africa does not allow for youth offenders to be monitored continuously and receive the necessary help to be fully rehabilitated.”*
Post‐release environment and lack of opportunities for youth offenders	5	P20: *“Youth offenders enter the same community where offending is a way of life and lack of opportunity for them results in reoffending.”* P34: *“The ability of an individual to be reintegrated functionally into society also is dependent on the community—are they willing to accept this individual back and give them the opportunities that would enable functional reintegration.”*
Inadequate judicial process	1	P13: *“The… justice system does not adequately allow for children who may not understand the discussion, or questions asked in courts… In addition if the offender is encouraged to develop his language and literacy skills, he/she may be more inclined to have less inclination to present with antisocial behaviour.”*

*Theme 3: Conditions required for successful reintegration*		
Necessary intervention and support services	7	P25: *“With the right support from professionals working with psychological challenges and trauma, youth can be given strategies and coping mechanisms.”* P27: “*With the right support and services they should be able to be reintegrated.”*
Need for specialised rehabilitation	4	P6: *“If the offender is encouraged to develop his language and literacy skills… may be more inclined to… present with antisocial behavior.”* P33: *“… offenses linked to communication and language aspects… when these needs are not attended to then it can exacerbate criminal activities.”*
Need for systemic reform	2	P13: *“… in a different system that prioritises rehab during incarceration could possibly aid in reintegration…”* P19: *“Rehabilitation would depend on many factors including: (the) protocol that is followed by the correctional centre…. Is there a social worker involved to assess the living conditions, family structure, etc., what support do they receive once released.”*

*Theme 4: Individual specific variables affecting potential for rehabilitation and reintegration*		
Specific offence committed	5	P19: *“What was the nature of the crime.”* P34: *“In some cases, I think that rehabilitation is possible, while in others, it is unlikely (e.g., perhaps rehabilitating a new gang initiate or a person who murdered as a means of self‐defence or stole out of desperation may be more likely than a rapist or serial killer).”*
Individual psychological barriers	2	P10: *“If there is a mental disability or psychological component it may not always be possible.”* P15: *“Another case after 5 years of cognitive therapy and group‐based interactions the individual shows no remorse or care and frequently shows enjoyment of others' pain and suffering. I agree it is possible to reintegrate but should be evaluated individually.”*
Scepticism regarding outcomes	3	P31: *“I am unsure because one can never be sure about the outcome of youth offenders being rehabilitated.”* P 32: *“A small percentage might be able to reintegrate.”*

*Note*. Some participants contributed to more than one theme thus the total frequency with which themes occurred was > 35.

The majority of participants (*n* = 29, 90.3%) agreed that societal attitudes contribute to the challenges that youth offenders face during reintegration into society, while one participant (3.2%) disagreed and two (6.5%) indicated that they were unsure. Participant age (*p* = 0.0.76, *r_s_* =  0.323), years’ experience (*p* = 0.068, *r_s_* =  0.332), CJS‐related training (*p* = 0.144, *r_pb_* = −0.268), and prior service delivery to youth offenders (*p* = 0.425, *r_pb_* = −0.160) showed no significant influence on this belief. Participants were also asked to identify societal attitudes they believed could hinder the reintegration process. Upon analysis, the dominant themes that emerged (Figure 1) were the stigma, labelling and stereotyping experienced by former youth offenders as dangerous [*“People from prisons are stigmatized.”* (P2); *“Labeling the offender as dangerous.”* (P4)], doubts about the possibility for change and rehabilitation [*“Believing they cannot change and will end up doing unlawful acts again.”* (P19); *“People often believe, once a criminal always a criminal.”* (P26); *“The perspective that an offender cannot change.”* (P27)], discrimination against former youth offenders driven by their criminal history [*“People would be apprehensive towards people who are coming out of jail. Getting jobs and forming relationships will be negatively affected.”* (P24); *“Bias and discrimination, lack of job opportunities, difficulty finding correct housing…”* (P30)], society's fear of youths reoffending and concerns about safety and the trustworthiness of former youth offenders [*“… closing doors of opportunities because of such labels.”* (P4); *“People would also likely be worried about safety and have preconceived notions regarding…”* (P30)], and additional bias that exists for those with speech, language or cognitive difficulties [*“… poor literacy and communication skills are stigmatic for low cognition and potential.”* (P7); *“… society may be less tolerant of offenders with speech impediments…”* (P13); *“People sometimes view persons with disabilities as incompetent and people can be less open to them due to a lack of knowledge.”* (P20)].

**FIGURE 1:**
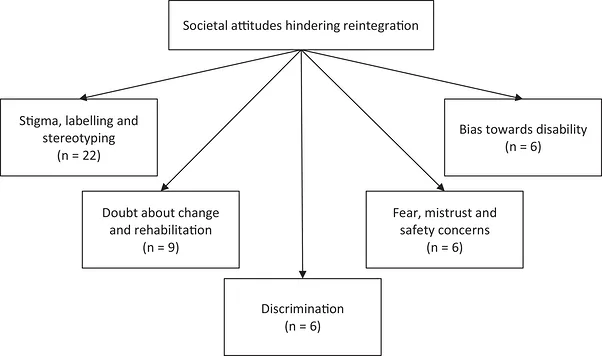
Perceived societal attitudes affecting youth offender reintegration (*n* = 31). *Note*: Some participants contributed to more than one theme thus the total frequency with which themes occurred was > 31.

The participants were then asked to explain what role they believe communication disorders could play in the behaviour and social interactions of youth offenders. Thirty‐three participants (94.3%) volunteered responses. The participants suggested that communication disorders contribute to emotional and behavioural difficulties due to difficulty expressing themselves, increasing the risk of aggression or criminal behaviour [*“Communication disorders can stir up many feelings of frustration… which could manifest as… acting violently”* (P3); *“Difficulty communicating their thoughts, needs and desires may result in frustration and conflict”* (P10)]. Participants also reported that communication difficulties often lead to social isolation and withdrawal [*“They may have poor pragmatic skills that result in them being bullied [or] isolated.”* (P10); *“Difficulties with language can lead to social withdrawal.”* (P17)] and negatively impact self‐esteem and confidence [*“It could affect their self‐belief, self‐confidence [and] self‐esteem”* (P26); *“It may lead to reduced self‐esteem and self‐worth”* (P28)]. Furthermore, communication disorders were seen as contributing to misunderstanding and conflict due to difficulty understanding social norms [*“Not understanding social norms and cues could lead to miscommunication…”* (P6)], and due to poor self‐expression [*“Escalation of potentially conflict‐laden situations.”* (P6); *“[If] the youth cannot communicate [their] needs and frustrations… may seek to act out.”* (P13)]. Participants also noted that communication impairments limit the education and employment opportunities of youth offenders, increasing their vulnerability and the risk of criminal involvement [*“Poor (communication skills) could result in diminished career opportunities, thus leading to crime.”* (P2); *“Due to stigma or difficulty communicating, it may also prevent them from securing employment.”* (P28)]. Additionally, some suggested that youth with communication difficulties are more susceptible to manipulation and peer pressure [*“Youth with communication disorders may do anything to belong to a group.”* (P11)]. Finally, participants indicated that communication disorders may hinder the participation of youth offenders in rehabilitation programs due to limited engagement and poor social interaction [*“It may even exacerbate the negative and bullying behaviours of other offenders… negatively impacting rehabilitation”* (P14); *“They would… not show interest in the rehabilitation process.”* (P31)].

To determine the participants’ perceptions of potential biological, environmental, and mixed risk factors influencing youth offending, they were presented with various risk factors and asked to rate the extent to which they believe each risk factor could contribute to youth offending behaviour (Table 2).

**TABLE 2:** Perceptual rating of risk factors contributing to youth engagement in offending behaviour (*n* = 31).

Risk factor	Disagree	Unsure	Agree
	n (%)	n (%)	n (%)
*Biological risk factors (n = 31)*			
Learning disorder	—	1 (3.2)	30 (96.8)
Attention‐deficit / Hyperactivity disorder (ADHD)	1 (3.2)	4 (12.9)	26 (83.9)
Acquired brain injury	2 (6.5)	3 (9.7)	26 (83.9)
Genetic predisposition	2 (6.5)	4 (12.9)	25 (80.6)
Autism spectrum disorder (ASD)	4 (12.9)	10 (32.3)	17 (54.8)
Late language emergence (LLE)	8 (25.8)	9 (29.0)	14 (45.2)
Physical impairment	8 (25.8)	9 (29.0)	14 (45.2)
Biological sex (male or female)	12 (38.7)	7 (22.6)	12 (38.7)
Hearing impairment (HI)	8 (25.8)	12 (38.7)	11 (35.5)
Preterm birth (PTB)	13 (41.9)	9 (29.0)	9 (29.0)
Low birth weight (LBW)	14 (45.2)	11 (35.5)	6 (19.4)
Visual impairment	12 (38.7)	13 (41.9)	6 (19.4)

*Environmental risk factors (n = 29)*			
Emotional abuse	—	—	29 (100.0)
Emotional neglect	—	—	29 (100.0)
Gang involvement	—	—	29 (100.0)
Living in high‐crime neighbourhoods	—	—	29 (100.0)
Sexual abuse	—	—	29 (100.0)
Verbal abuse	—	—	29 (100.0)
Parental psychopathology	—	1 (3.4)	28 (96.6)
Physical abuse	—	1 (3.4)	28 (96.6)
Physical neglect	—	1 (3.4)	28 (96.6)
Social neglect	1 (3.4)	—	28 (96.6)
Poverty	—	2 (6.9)	27 (93.1)
Poor academic performance	1 (3.4)	4 (13.8)	24 (82.8)
Parental divorce	2 (6.9)	6 (20.7)	21 (72.4)
Maternal education	3 (10.3)	10 (34.5)	16 (55.2)
Paternal education	3 (10.3)	10 (34.5)	16 (55.2)
Maternal occupation	5 (17.2)	9 (31.0)	15 (51.7)
Paternal occupation	5 (17.2)	9 (31.0)	15 (51.7)
Birth order (being younger or older)	6 (20.7)	17 (58.6)	6 (20.7)

*Mixed risk factors (n = 29)*			
Domestic conflict	—	2 (6.9)	27 (93.1)
Poor mother‐child attachment	—	2 (6.9)	27 (93.1)
Poor mother‐child interaction	—	3 (10.3)	26 (89.7)
Poor father‐child interaction	—	2 (6.9)	27 (93.1)
Poor father‐child attachment	—	4 (13.8)	25 (86.2)

Participants without prior training to work with individuals in the CJS were significantly more likely to agree that a genetic predisposition (*p* = 0.048, *r_pb_* = 0.358), a diagnosis of ASD (*p* = 0.016, *r_pb_* = 0.430), a history of sexual abuse (*p* = 0.030, *r_pb_* = 0.404), parental psychopathology (*p* = 0.038, *r_pb_* = 0.388), and domestic conflict (*p* = 0.007, *r_pb_* = 0.492) could contribute to youth engaging in criminal activity than those who did receive such training. Similarly, participants with no previous experiences with service delivery to youth offenders were more likely to agree that gang involvement could contribute to youth engaging in criminal activity than participants with prior experience (*p* = 0.011, *r_pb_* = 0.491).

## 4 Discussion

South African SLTs generally expressed positive attitudes towards youth offenders and their potential for rehabilitation. Just over half of the participants (*n* = 21, 60.0%) agreed that youth offenders can be successfully rehabilitated and reintegrated into their communities. Although there is currently a lack of literature investigating the perceptions of HCPs towards youth offenders specifically, existing research suggests that public opinion toward youth offender rehabilitation is generally positive (Moon et al. 2003; Welch et al. 2019). This may indicate that HCPs, such as SLTs, could hold similar positive views towards the population. However, education and training had no significant impact on participants’ perceptions about rehabilitation and reintegration. This contrasts with previous research, which found that HCPs with higher levels of education demonstrate significantly more positive attitudes towards offenders (Martinaki et al. 2025) and that people in the community who completed some tertiary education generally perceived the rehabilitation potential of youth offenders more favourably (Baker et al. 2014). Because education is known to generally improve social tolerance towards other groups (Lee 2024), further research could prove valuable to investigate what relationships exist between level of education and the perceptions of South African SLTs towards the population. Despite the limited availability of SLT services for youth offenders in South Africa, the positive attitudes demonstrated by SLTs towards the rehabilitation and reintegration of youth offenders are encouraging, as it reflects a general openness towards the population. This favourable disposition may provide a strong foundation for advocacy efforts aimed at expanding SLT service delivery within youth justice settings. In particular, it suggests that the profession may be responsive to increased involvement in the assessment and rehabilitation of youth offenders, thus supporting the integration of SLTs into youth offender rehabilitation teams.

When asked to elaborate on their beliefs, participants emphasised that the potential of a youth offender to be rehabilitated and reintegrated is highly variable and dependent on the underlying cause of the criminal behaviour, the type of crime committed, the systemic barriers posed by both the CJS as well as the structural support provided in the post‐release environment. Participants’ beliefs are supported in literature which found that intra‐organisational factors, such as those within the juvenile justice and law enforcement systems (O'Neill et al. 2017) and the post‐release environment (O'Neill et al. 2017; Saadati and Saadati 2023) could negatively affect youth offender rehabilitation and reintegration. Additionally, participants’ consideration of the cause of the criminal behaviour could potentially also show good insight into a personal barrier to rehabilitation and reintegration. For instance, research has found that a history of substance use or coming from a socioeconomically disadvantaged background could increase a youth offender's risk of reoffending (Özbek et al. 2025; Shinkfield and Graffam 2009; Skjærvø et al. 2024). Similarly, children reintegrated into communities where they have access to substances may still be at risk of reoffending, given that they have not yet reached full cognitive, emotional and intellectual maturity and are therefore at higher risk of reengaging in high risk behaviour (Özbek et al. 2025). These findings indicate that the participants have relatively good insight into the environmental barriers faced by youth offenders during their reintegration. This insight could prove critical for the planning of functional therapeutic outcomes during SLT service delivery. Further research is, however, needed to explore how SLTs could support youth offenders in this regard, particularly given the service delivery challenges reported by SLTs working in youth correctional contexts (Martin et al. 2019).

The participants’ identification of the important role of the community post‐release (Table 1) is especially relevant since youth offenders are more likely to reoffend if not well supported by their family unit and larger community during reintegration (UNODC 2012). The community does, however, require sufficient resources and social support to perform this function (UNODC 2012). This could prove challenging in the South African context where, as the participants also highlighted, they experience resource constraints, lack sufficient follow‐up and support services, and lack appropriate infrastructure (Chikadzi 2017; Nyathi et al. 2024). Further research is therefore needed to investigate how South African youth offender rehabilitation programmes could rise to the challenge of supporting youth offender reintegration through adequate preparation for life post‐release, as well as to foster a sense of openness and responsibility among communities to support youth offenders in their reintegration into society (Singh 2016). Additionally, the role of SLTs in preparing youths for this reintegration through communication intervention needs to be explored.

Most participants (*n* = 29, 90.3%) agreed that societal attitudes could impede the successful reintegration of youth offenders through stigmatisation, labelling and stereotyping. These findings indicate that reintegration may not only be constrained by the youth offenders’ capacity for rehabilitation, but also by society's willingness to recognise and support behavioural change. The prominence of stigma and labelling is consistent with labelling theory, whereby deviant labels may contribute to stigmatisation, marginalisation and reduced opportunities for prosocial participation (Cronje 2025). This is particularly relevant to participants’ perceptions that former youth offenders may be denied employment, housing and social opportunities because of their criminal history, as negative societal attitudes such as these have been associated with poorer reintegration outcomes and increased recidivism (Schneider and Weber 2020). Therefore, the findings suggest that participants believe that persistent societal labelling could reinforce barriers to reintegration even after a young person has completed their rehabilitation process. These findings have important implications for professional reflection among HCPs, including SLTs. HCPs should critically consider whether societal assumptions about offending may influence their own expectations of a young person's capabilities, motivation or potential for change. This is particularly important given participants’ perceptions that speech, language and cognitive difficulties may themselves be stigmatised by society. There is, in fact, a basis for this perception as research has already established that other youth justice staff working in youth correctional centres perceives communication difficulties among youth offenders as behavioural difficulties (Perrotti et al. 2026). HCPs working with youth offenders should therefore adopt intervention approaches that recognise these contextual factors, promote participation and agency, and minimise the risk of reinforcing stigma. For SLTs, this could include supporting communication, self‐efficacy, social connectedness and participation through interventions that build resilience among youth offenders while still remaining within their professional scope, as suggested by Rothbart and Sohlberg (2021). Adopting such approaches could help, not only support youth offenders’ resilience when released from custody, but also to optimise clinical outcomes in SLT intervention, as the intervention is more person‐centered and holistic (Rothbart and Sohlberg 2021). Further research is needed to explore how HCPs, such as SLTs, can actively foster resilience in youth offenders by promoting belonging, learning, coping and identity development throughout the rehabilitative process (Stubbs and Hart 2020) as well as to explore the efficacy of resilience‐focused rehabilitation provided by SLTs working in youth justice settings. This knowledge could help enable functional intervention practices, which could help to reduce the harmful effects of labelling and stigmatisation towards youth offender's post‐release, thereby supporting reintegration and reducing recidivism in the population.

Participants reported that communication disorders negatively affect youth offenders’ behaviour and social participation by contributing to emotional and behavioural difficulties. Such disorders limit a child's ability to effectively express their needs, make choices, and develop social competencies (Schneider and Goldstein 2008), impairing socio‐emotional functioning, quality of life, and communicative participation (Duinmeijer et al. 2025). Adolescents who experience difficulty communicating often experience frustration, leading to peer conflict and behavioural challenges when their attempts to communicate fail (Jansen et al. 2020). This frustration has been linked to aggression and antisocial behaviour, which can intensify social withdrawal and isolation (Pickles et al. 2016; Stuewig et al. 2015; Xue et al. 2025). Social exclusion, in turn, limits opportunities to learn social language and modelled interactions, further straining relationships and hindering prosocial communication (Helland et al. 2014; Xue et al. 2025). As participants suggested, these difficulties often stem from a limited understanding of social norms and a limited ability to express oneself constructively (Table 1). This cycle of communicative breakdown may also reduce academic engagement, impede literacy development, and increase the risk of offending behaviour (Hopkins and Clegg 2022). Youth offenders with communication difficulties thus face a dual disadvantage: the stigma associated with their criminal record, combined with poor language and literacy skills, further reduces their educational attainment and employment prospects (Deakin et al. 2020; Macharey and Von Suchodoletz 2008). This is despite research highlighting that employment among youth offenders supports the development of a more coherent prosocial identity, potentially due to reduced social exclusion (Oswald 2022). This perspective was also highlighted by the participants who expressed the beliefs that youth offenders frequently face a lack of sufficient or appropriate employment opportunities due to stigma related to their potential communication disorder or their status as an offender. This shows good insight among the participants as an inability to secure employment after incarceration could therefore be detrimental to the rehabilitation process and contribute to recidivism amongst the youth offender population.

Similar to what is suggested in literature (Bryan et al. 2015; Winstanley et al. 2020), the participants also indicated that youth offenders with communication disorders are unable to effectively participate in rehabilitation programmes, reducing the effectiveness of these programmes and increasing their risk of recidivism. The participants’ perceptions of the challenges faced by youth offenders with communication disorders are therefore relatively well informed as these difficulties are known to be commonly observed in the population, including reduced participation in social and academic contexts (Abhishek and Balamurugan 2024; Lankester et al. 2025), increased antisocial behaviour (Carroll et al. 2023), exclusion and self‐isolation (Mishra and Biswal 2018), as well as reduced employment prospects (Oswald 2022).

When asked to rate their level of agreement with various risk factors which could potentially contribute to youth offending behaviour, participants without training to work with youth offenders were more likely to attribute criminal behaviour to biological factors, such as genetics and ASD, as well as personal factors such as a history of sexual abuse. While children with neurodevelopmental disorders (NDDs) are overrepresented in the CJS (Hughes et al. 2017a), understanding how NDDs may increase children's risk of justice system involvement, rather than directly causing criminal behaviour (Bartol and Bartol 2017), requires a more nuanced exploration. For instance, while limited, literature has found offending behaviour of people with ASD to be related to ASD characteristics such as idiosyncratic beliefs and behaviours without consideration of social consequences (Helverschou et al. 2018). The literature is clear that there is no single gene or set of genes that directly predisposes someone to engage in criminal behaviour, and that biological factors alone have limited to no influence on criminality (Maree 2024; Shoemaker 2018). There are, however, certain genetic bundles which may increase someone's vulnerability to behaviours associated with offending (Maree 2024). They may therefore be predisposed towards characteristics such as impulsivity, aggression and poor emotional regulation which, when combined with social or environmental factors such as unemployment or living in a disorganised community, can increase the likelihood of engaging in criminal activity (Maree 2024). Criminal behaviour is thus widely recognised by experts to be a complex interplay between an individual's biology and their environment. However, due to the phrasing used in this study's survey, participant responses could potentially be open to different interpretations. This issue is examined in more detail in the study limitations. Additionally, the phrasing of the question asking participants to rate various risk factors limited the study's ability to determine their perceptions of the degree to which these risk factors influence youth offender rehabilitation. Further research should explicitly examine SLTs’ perceptions of these influences.

The minority of participants who had received additional training on youth offenders were less likely to attribute criminal behaviour to biological factors. This may reflect a more comprehensive understanding of the complex and multifaceted causes of offending behaviour, highlighting the potential value of education and training regarding youth offenders for SLTs. Regardless of whether SLTs attribute youth offending behaviour to biological factors, biology plays a role to some extent, as discussed above and is often attributable to neurodevelopmental conditions which affect cognition and behaviour. (Flannigan et al. 2019; Hughes et al. 2017a). Given that certain cognitive and behavioural conditions may increase the risk of offending behaviour (Maree 2024), SLTs could play an important role in rehabilitation by supporting youth offenders’ communication and cognitive‐linguistic development. Strengthening these skills may enhance their ability to regulate their behaviour, navigate social interactions, and express themselves more constructively. However, this perspective was not directly investigated in this study. Future research should explore the relationship between professional training and beliefs regarding the cause of criminal behaviour in greater depth.

Similarly, while research has found that a history of sexual abuse in children increases their likelihood of engaging in offending behaviour, the offences committed by these children are potentially linked to behavioural and psychological difficulties commonly experienced by children who have been sexually exploited (Cockbain and Brayley 2012). Thus, experiencing abuse or maltreatment could contribute to psychological distress or trauma responses, which may, in turn, contribute to criminal behaviour in children due to maladaptive coping mechanisms or mental health difficulties (Perez et al. 2018). Additional research is therefore also needed to investigate the participants’ understanding of the nuanced relationships between NDDs, parental psychopathology, sexual abuse, domestic conflict, and gang involvement and youth offending.

Youth offenders represent a “double at‐risk” population due to their age and “offender” status (Ahmed Shafi 2020), facing greater vulnerability to stigma and discrimination (Deakin et al. 2020) as well as more severe and comorbid health conditions (Lennox et al. 2014), including communication disorders. Despite this, there is currently limited literature exploring HCPs’ perceptions of offenders, and an even greater lack of research focusing on youth offenders, aside from a study involving French juvenile justice social workers (De Boer et al. 2025). The need for such research is particularly pressing in contexts such as South Africa, where youth offenders are entitled to comprehensive healthcare services that facilitate participation in rehabilitation programmes (DCS 2005). This knowledge could potentially inform the design of education and training programmes for HCPs, including continuing professional development, enabling them to reflect on how they access, assess, and provide services to this population.

### 4.1 Limitations

In this study, the authors faced a few limitations. The sample size included in this study is relatively small, which limited statistical power and meant that only large effect sizes (0.5) could be detected with sufficient reliability. Another limitation of this study relates to the inability to assess potential non‐response bias. Although demographic data were collected at the beginning of the survey, eight responses were excluded as these respondents completed only minimal biographical information and did not proceed to the substantive survey items, with several providing responses to only one or two items before discontinuing. Within the retained sample, some missing data were present across variables and were addressed using pairwise deletion. Consequently, a clear distinction between completers and non‐completers could not be established, precluding meaningful comparisons to assess potential non‐response bias. Additionally, the perspective of this study is limited to only a single professional population and geographic location. Thus, given the aforementioned limitations, the generalisability of the study's findings is limited. While the findings of this study will still provide noteworthy preliminary insights into the perceptions of HCPs, specifically SLTs in South Africa, regarding youth offenders, future research should focus on expanding this research by obtaining a larger sample size and including various HCPs such as nurses, doctors, dentists, and mental health professionals who are involved in youth offender health and rehabilitation teams. It is also recommended that research on this topic be done in various contexts, such low‐ and high‐income countries, to obtain a more holistic overview of the perspectives of HCPs towards youth offenders and their rehabilitation. Additionally, replicating this study in contexts where SLT services are established within youth justice settings may yield more specific insights into SLTs’ perspectives on youth offenders and their rehabilitation.

Future research on this topic can expand on the methodological robustness of the study by exploring hybrid methodologies, such as combining an online interview after participants have completed the online survey, in order to obtain further depth and insight into their perceptions. The survey used in this study can also be further adapted for future research. For example, the section of the survey that explores participants’ perceptions of the risk factors contributing to youth offending behaviour can be adapted to include qualitative responses explaining how or why participants believe a specific risk factor could contribute to youth offending behaviour. Additionally, participants could be provided with a text box to enter risk factors not listed in the survey. This could help to obtain a more nuanced understanding of the participants' perceptions of which risk factors “contribute to” or “cause” youth to engage in criminal behaviour as well as how.

### 4.2 Conclusions

Participants generally held positive views towards youth offenders and their potential for rehabilitation and reintegration. The participants identified several individual, environmental and social variables which could affect the rehabilitation in reintegration processes of youth offenders. This is a promising finding as it shows that the participants already have some degree of insight into the needs of youth offenders. Participants also demonstrated good insight into the potential adverse effects the presence of a communication disorder could have for a youth offender, chief among them the inability to functionally participate in rehabilitation programmes, increasing their risk of recidivism. Education and training had a significant impact on the participants’ likelihood of attributing criminal behaviour among youth offenders to biological factors. While this study did not investigate this relationship in depth, it requires further exploration. Future research is needed to obtain insight into the perceptions of various HCP disciplines functioning in different contexts towards youth offenders and their rehabilitation.

## Funding

The authors have nothing to report.

## Conflicts of Interest

The authors declare no conflicts of interest.

## Ethics Statement

This study obtained IRB clearance from the research ethics committee of the Faculty of Humanities at the University of Pretoria (*HUM005/0324*)

## Supporting information




**Supporting Information**: jlcd70345‐sup‐0001‐SuppMat.docx

## Data Availability

The dataset analysed in the current study is available on the University of Pretoria's Figshare Research Data Repository and can be accessed using the following link: https://doi.org/10.25403/UPresearchdata.29924987.

## Endnotes

1 In South Africa, youths are deemed young people aged 13 to 18 years. However, in special circumstances, young people between 18 and 25 years are deemed “youth” by the DCS (Department of Correctional Services [DCS] 2005).
