# Supplementary material for: South African Speech‐Language Therapists’ Perceptions of Youth Offenders and Youth Offender Rehabilitation
Source: Int J Lang Commun Disord. 2026 Sep 30;61(6):e70345. doi: 10.1111/1460-6984.70345 (PMC13625664; doi:10.1111/1460-6984.70345)
Supplement: Supplementary file 1 [file JLCD-61-0-s001.docx]

**Supplemental material.** Survey

**Perceptions of South African speech-language pathologists on their role in youth correctional facilities and youth offender rehabilitation**

A. **Demographic Information**
1. **Are you registered with the Health Professions Council of South Africa as a speech-language pathologist (SLP)?**

1 - Yes

2 - No

2. **Are you currently practising as a SLP in South Africa?**

1 - Yes

2 - No

3. **What is your age in years?** *(Please type the number only)*

_____ years

4. **Please indicate your gender:**

1 - Male

2 - Female

3 - Non-binary/third gender

4 - Prefer not to say

5 - Other (please specify): ______________

5. **What is your highest level of education completed?**

1 - Professional/qualifying bachelor’s degree (4 years)

2 - Master’s degree

3 - Doctoral degree

4 - Postdoctoral research fellowship

5 - Other (please specify): ______________

6. **How many years of experience do you have working as a SLP?** *(Please type the number only)*

_____ years

7.1 **Have you received any training in working with any persons in the criminal justice system?**

1 - yes

2 - no

3 - unsure

**7.2 If you answered “yes” to question 7.1, please briefly describe the nature of the training.**

____________________________________________________________________________________________________________________________________________________________

8.1 **Have you ever provided any SLP services to a youth offender in the criminal justice system? In South Africa, a youth offender is defined as a person between the ages of 14 and 25 years old.**

1 - yes

2 - no

3 - unsure

**8.2 How frequently have you delivered services to a youth offender in the criminal justice system?**

1 - infrequently

2 - somewhat infrequently

3 - unsure

4 - somewhat frequently

5 - frequently

**8.3 In which context were these services delivered?** *(please select all that apply)*

1 - schools

2 - hospitals

3 - correctional facilities

4 - private practice

**8.4 What were the patients’ age in years?** *(please select all that apply)*

1 - 14 to 15 years

2 - 16 to 17 years

3 - 18 to 19 years

4 - 20 to 21 years

5 - 22 to 23 years

6 - 24 to 25 years

**8.5 What difficulties or disorder(s) did these patients present with?**

____________________________________________________________________________________________________________________________________________________________

B. **Perceptions regarding youth offenders and youth offender rehabilitation**
   1.1 **Indicate your level of agreement with the following statement:**

**“*I am of the opinion that youth offenders can be rehabilitated and functionally reintegrated into society.*”**

1 - strongly disagree

2 - disagree

3 - unsure

4 - agree

5 - strongly agree

**1.2 Please expand on the reason(s) for your opinion stated above:**

____________________________________________________________________________________________________________________________________________________________

2.1 **Indicate your level of agreement with the following statement:**

**“*I believe that SLPs can positively impact the rehabilitation of youth offenders.*”**

1 - strongly disagree

2 - disagree

3 - unsure

4 - agree

5 - strongly agree

2.2 **Please expand on the reason(s) for your belief stated above:**

____________________________________________________________________________________________________________________________________________________________

3.1 **How comfortable do you feel potentially working with youth offenders in a correctional facility?**

1 - not comfortable at all

2 - uncomfortable

3 - unsure

4 - comfortable

5 - very comfortable

3.2 **What makes you comfortable or uncomfortable with the idea of working with youth offenders in a correctional facility or youth care centre?**

____________________________________________________________________________________________________________________________________________________________

4.1 **Indicate your level of agreement with the following statement:**

**“*I believe that the identification and intervention of communication disorders by SLPs can contribute to preventing youth offenders from reoffending in the future.*”**

1 - strongly disagree

2 - disagree

3 - unsure

4 - agree

5 - strongly agree

4.2 **Please expand on the reason(s) for your level of agreement as stated above:**

____________________________________________________________________________________________________________________________________________________________

5 **In your opinion, what role could communication disorders play in youth offenders’ behaviour and social interactions?**

____________________________________________________________________________________________________________________________________________________________

6.1 **Indicate your level of agreement with the following statement:**

**“*I believe that societal attitudes contribute to the challenges youth offenders could face during reintegration into society.*”**

1 - strongly disagree

2 - disagree

3 - unsure

4 - agree

5 - strongly agree

**6.2 In your opinion, which societal attitudes could potentially prove challenging for youth offenders who are attempting to reintegrate into society?**

____________________________________________________________________________________________________________________________________________

7. **Please rate your level of agreement with the following statements:**

1 = strongly disagree	2 = disagree	3 = unsure	4 = agree	5 = strongly agree

***“The following biological risk factors might contribute to youth engaging in criminal activity:”***

Low birth weight (LBW)	1	2	3	4	5

Preterm birth (PTB)	1	2	3	4	5

Genetic predisposition	1	2	3	4	5

Attention-Deficit/Hyperactivity Disorders (ADHD)	1	2	3	4	5

Autism Spectrum Disorder (ASD)	1	2	3	4	5

Hearing impairment	1	2	3	4	5

Visual impairment	1	2	3	4	5

Physical impairment	1	2	3	4	5

Late talker	1	2	3	4	5

Learning disorders	1	2	3	4	5

Acquired brain injury	1	2	3	4	5

Biological sex (male or female)	1	2	3	4	5

***“The following environmental risk factors might contribute to youth engaging in criminal activity”***

Emotional neglect	1	2	3	4	5

Social neglect	1	2	3	4	5

Physical neglect	1	2	3	4	5

Verbal abuse	1	2	3	4	5

Physical abuse	1	2	3	4	5

Emotional abuse	1	2	3	4	5

Sexual abuse	1	2	3	4	5

Poverty	1	2	3	4	5

Paternal education	1	2	3	4	5

Maternal education	1	2	3	4	5

Paternal occupation	1	2	3	4	5

Maternal occupation	1	2	3	4	5

Birth order (being younger or older)	1	2	3	4	5

Parental divorce	1	2	3	4	5

Parental psychopathology	1	2	3	4	5

Poor academic performance	1	2	3	4	5

Living in high-crime neighbourhoods	1	2	3	4	5

Gang involvement	1	2	3	4	5

***“The following mixed risk factors might contribute to youth engaging in criminal activity”***

Poor mother-child interaction	1	2	3	4	5

Poor mother-child attachment	1	2	3	4	5

Poor father-child interaction	1	2	3	4	5

Poor father-child attachment	1	2	3	4	5

Domestic conflict	1	2	3	4	5

C. **Perceptions regarding the possible role of SLPs in youth offender rehabilitation**
1. **Indicate your level of agreement with the following statement:**

***“I am familiar with the potential role of SLPs in youth offender rehabilitation.”***

1 - Not familiar at all

2 - somewhat unfamiliar

3 - unsure

4 - somewhat familiar

5 - very familiar

2. **Please indicate your level of agreement with the following statement:**

1 = strongly disagree	2 = disagree	3 = unsure	4 = agree	5 = strongly agree

***“SLPs could potentially fulfil the following roles and responsibilities in youth offender rehabilitation programmes:”***

Speech and language screening	1	2	3	4	5

Hearing screening	1	2	3	4	5

Communication assessment	1	2	3	4	5

Individual therapy	1	2	3	4	5

Group therapy	1	2	3	4	5

Support literacy development and acquisition	1	2	3	4	5

Youth care centre staff education and training	1	2	3	4	5

Family education and training	1	2	3	4	5

Social communication skills development	1	2	3	4	5

Support reintegration in community	1	2	3	4	5

Support communication skills development for future employment

1	2	3	4	5

Assist in developing rehabilitation programmes	1	2	3	4	5

3. **What resources or training do you believe would enhance the effectiveness of SLPs when working with youth offenders?** *(please select all that apply)*

1 - training on specific communication disorders and needs prevalent in this population

2 - workshops on understanding the causes of youth offending

3 - collaboration with other rehabilitation professionals working with youth offenders

4 - access to relevant assessment tools and materials

5 - professional ethics and practice guidelines

6 - other (please specify): ______________

**4.1 Indicate your level of agreement with the following statement:**

**“*I believe there is a need for increased advocacy for the inclusion of SLPs in youth offender rehabilitation programmes.*”**

1 - strongly disagree

2 - disagree

3 - unsure

4 - agree

5 - strongly agree

**4.2 Please briefly expand on the reason(s) for your belief stated above.**

____________________________________________________________________________________________________________________________________________________________

5. **In your opinion, what potential barriers exist for SLPs to effectively contribute to youth offender rehabilitation in South Africa?**

____________________________________________________________________________________________________________________________________________________________

6. **Please share any other thoughts or needs that you might have on the role of SLPs when working with youth offenders.**

____________________________________________________________________________________________________________________________________________________________

**Thank you for your participation in this study and for completing this survey.**
